# Postbiotics and their biotherapeutic potential for chronic disease and their feature perspective: a review

**DOI:** 10.3389/frmbi.2025.1489339

**Published:** 2025-03-03

**Authors:** Zerihun Asefa, Abera Belay, Eyuel Welelaw, Meseret Haile

**Affiliations:** ^1^ Food Science and Nutrition Research, Ethiopian Institute of Agricultural Research, Holeta Agricultural Research Center, Holeta, Oromia, Ethiopia; ^2^ Department of Food Science and Applied Nutrition, Bioprocessing and Biotechnology Center of Excellence, Addis Ababa Science and Technology University, Addis Ababa, Ethiopia; ^3^ Department of Food Science and Postharvest Technology, Wachemo University, Wachemo, Ethiopia

**Keywords:** biotherapeutic, biofunctional, microbiome, chronic disease, bioactive

## Abstract

Postbiotics, which are bioactive compounds derived from the metabolic processes of probiotics, are gaining recognition as a promising alternative for managing chronic diseases without the need for live microorganisms, positioning them as a valuable strategy in biotherapeutics that offers both curative and preventive techniques in modern medicine. This paper provides a comprehensive review of the potential health benefits of postbiotics, particularly concerning noncommunicable diseases like diabetes, cancer, obesity and cardiovascular conditions, which present significant global health challenges. We explore the various mechanisms by which postbiotics exert their beneficial effects, including immune modulation to enhance the body’s immune response and reduce inflammation, as well as improving gut barrier function to maintain gut integrity and prevent increased intestinal permeability. Additionally, the antioxidant properties of postbiotics play a critical role in neutralizing oxidative stress, which is linked to the progression of chronic diseases. Despite the encouraging insights into their health benefits, we highlight the urgent need for further research to clarify the specific roles of different postbiotic components. A deeper understanding of these mechanisms is essential for developing targeted preventive healthcare applications, and by advancing this knowledge, we aim to create innovative strategies that could significantly enhance health outcomes for at-risk populations. Ultimately, integrating postbiotics into health interventions has the potential to improve preventive care and contribute to the overall well-being of affected individuals and communities.

## 1 Introduction

Postbiotics are bioactive compounds that are derived from the metabolic byproducts of probiotics through the fermentation process. They are non-viable bacterial products or metabolic byproducts from probiotic microorganisms that have biologic activity in the host. It includes short-chain fatty acids (SCFAs), exopolysaccharides (EPS), bioactive peptides (BAPs), cell components, organic acids, cell fragments, and vitamins, have potential health benefits such as anti-inflammatory, antioxidant, anti-cancer, and antihypertensive properties (Fattahi et al., 2020; Kamiloglu et al., 2022; Du et al., 2024). The term “postbiotic” is relatively new in the field of microbiome research and is used to describe these substances that confer health benefits similar to or distinct from those associated with probiotics. Postbiotics, as defined by the International Scientific Association of Probiotics and Prebiotics (ISAPP), are “preparations of inanimate microorganisms and/or their components that confer a health benefit on the host” (Salminen et al., 2021). A key aspect of this definition is that the final postbiotic product must include inactivated microbial cells or their components, with or without associated metabolites. Importantly, the definition excludes substantially purified metabolites in the absence of cellular biomass. For example, isolated compounds such as butyric acid or lactic acid should be referred to using their chemical nomenclature rather than being classified as postbiotics. They differ from probiotics, live microorganisms, and can include inactivated microbial cells or their metabolites. The concept of postbiotics gained attention as potential alternatives to probiotics, as they could potentially overcome some of the limitations associated with the use of live microorganisms’ probiotics. Probiotics are live microorganisms that confer health benefits when consumed in adequate amounts, have been extensively studied and applied in various areas of health. They are often used to alleviate symptoms associated with irritable bowel syndrome and to rebalance the gut microbiome following antibiotic use (Boyte et al., 2023; Shi et al., 2024). Although significant health benefits, there are some limitations associated with their viability and stability of the live microorganisms during storage and transit through the gastrointestinal tract (Shah, 2010). The viability of probiotics is a critical factor, as they need to be alive to confer health benefits. This requirement makes them sensitive to storage conditions, including temperature and pH levels, which can affect their efficacy over time (Scarpellini et al., 2021; Zhang et al., 2022).The beneficial effects of probiotics are not solely attributed to the live microorganisms themselves, but also to the metabolites, cellular components, and other byproducts produced by these microbes during fermentation. These non-viable microbial components and their metabolites i.e. postbiotics are generally more stable and resilient than live probiotics, as they are not affected by environmental factors or the host’s gastrointestinal conditions (Scarpellini et al., 2021; Zhang et al., 2022). Postbiotics can be more easily standardized and incorporated into various food, beverage, and pharmaceutical products, compared to the challenges of maintaining the viability of probiotic strains.

Noncommunicable diseases (NCDs) are a significant global public health issue, contributing to an estimated 74% of deaths in 2020, as noted by Jastrząb, Graczyk (Jastrząb et al., 2021) and Park, Joung (Park et al., 2022). Cardiovascular diseases, cancer, chronic kidney diseases, obesity, and diabetes are common NCDs, and their burden is expected to rise in the coming years due to urbanization, population aging, and lifestyle changes (Behera, 2020; Stasi et al., 2022). In 2020, cardiovascular disease was the primary cause of death with 17.9 million deaths, followed by cancer with 9.3 million deaths (Thorakkattu et al., 2022). In low- and middle-income countries, the high cost of treatment hinders access to care for NCDs. The treatment cost varies depending on the disease’s severity, type, and healthcare resources available in the country, making the cost of treating NCDs in LMICs much higher than in high-income countries, which creates significant barriers to treatment (Kundu et al., 2018; Njuguna et al., 2020). The cost of treating hypertension and diabetes in LMICs ranges between US$100 and US$500 and US$200 and US$1000 per year, respectively, while cancer treatment can cost up to US$10,000 per year, making access to care a major challenge for people in LMICs (Kundu et al., 2018; Njuguna et al., 2020).

## 2 Current chronic disease management and postbiotics

Postbiotics have the potential to enhance the efficacy of conventional therapies by modulating inflammation, improving gut health, and providing synergistic effects with medications (Scott et al., 2022; Wang S. et al., 2024). Chronic diseases are often associated with low-grade inflammation, and postbiotics can help regulate inflammatory responses by promoting the production of anti-inflammatory cytokines and inhibiting pro-inflammatory markers (Koshiyama, 2010; Mundula et al., 2022). This modulation can improve patient outcomes when postbiotics are administered alongside traditional treatments, such as pharmacotherapy.

Many conventional therapies disrupt gut microbiota balance, resulting in gastrointestinal side effects and dysbiosis. Postbiotics offer a promising solution by restoring gut microbiome, alleviating symptoms associated with antibiotics or other medications, and enhancing overall treatment adherence (Zhang et al., 2018). Beyond mitigating side effects, postbiotics play a critical role in modulating gut microbiota composition and influencing drug pharmacokinetics by enhancing absorption, metabolism, and therapeutic efficacy. For instance, microbial metabolites have been shown to increase the bioavailability of omeprazole by 269.9% through the modulation of cytochrome P450 enzymes (Zhang et al., 2024). Additionally, short-chain fatty acids (SCFAs) such as butyrate, acetate, and propionate lower intestinal pH, which improving drug solubility and absorption (Blaak et al., 2020; Liu et al., 2021). This mechanism has been demonstrated to enhance the bioavailability of drugs like lurasidone by 4.3-fold (Collins et al., 2024). Moreover, in cancer treatment, postbiotics exhibit anti-proliferative and anti-inflammatory properties that can moderate the effectiveness of conventional therapies while reducing adverse effects (Rad et al., 2020). These highlight the potential of postbiotics to enhance drug efficacy and minimize therapy-related complications.

Dietary interventions play a critical role in managing chronic diseases such as obesity, diabetes, and cardiovascular disease. Incorporating postbiotics into dietary strategies can significantly enhance their effectiveness by promoting a balanced gut microbiome and improving gut barrier function (Ozma et al., 2022; Li et al., 2024). Postbiotics can also influence the metabolism of dietary components by modulating gut microbiota composition.

The diversity of gut microbiota is linked to the fermentation of dietary fibers, which is essential for SCFA production, particularly butyrate, acetate, and propionate (Salamone et al., 2021; Maiuolo et al., 2024). This synergy between postbiotics and dietary approaches underscores the need for a holistic strategy in chronic disease management.

Fermented foods, such as yogurt, kefir, sauerkraut, kimchi, miso, tempeh, and kombucha, are among the richest sources of postbiotics (Darwish et al., 2022; Gill and Staudacher, 2023; Gurunathan et al., 2023). These foods not only provide beneficial microorganisms but also bioactive compounds generated during fermentation, supporting gut health and overall well-being (Beshkova and Pavlov, 2012; Darwish et al., 2022). Incorporating fermented foods into the diet can be a valuable strategy for enhancing postbiotic intake.

While postbiotics complement traditional therapies, they also hold considerable potential as standalone treatments for promoting human health and managing chronic diseases (Nagarajan et al., 2022; Freitas et al., 2023; Sorrenti et al., 2023). Certain microbial metabolites, such as SCFAs, bioactive peptides, and exopolysaccharides, positively influence metabolic health and reduce the risk of conditions like obesity, type 2 diabetes, and cardiovascular disease (Wu et al., 2023; Eslami et al., 2024). Postbiotics improve metabolic health by regulating lipid profiles, enhancing insulin sensitivity, and modulating immune responses, offering a balanced approach that reduces reliance on pharmacological interventions.

In gastrointestinal health, postbiotics have demonstrated efficacy in managing disorders like irritable bowel syndrome (IBS) and inflammatory bowel disease (IBD) by restoring gut barrier function and promoting the synthesis and assembly of tight junction proteins, which are essential for maintaining the structural integrity of the gut epithelium (Scott et al., 2022) and reducing inflammation ( (Valera et al., 2009). Additionally, they bolster immune function, particularly in individuals with compromised immune systems or chronic inflammatory conditions (Mehta et al., 2023).

### 2.1 Components of postbiotics

Postbiotics, comprising various constituents produced during the fermentation process or molecules that are found in the cell walls of some bacteria like teichoic acid, EPS, BAPs), and SCFAs such as butyrate, acetate, and propionate, are organic acids produced during the fermentation of dietary fibers by probiotic bacteria. Antimicrobial bacteriocins, BAPs, teichoic acids, and vitamins have demonstrated bioactive properties, including immunomodulation, anti-inflammatory, anti-microbial, anti-oxidant, and anti-proliferation (Scott et al., 2022; Vinderola et al., 2022). Studies by Mayorgas, Dotti (Mayorgas et al., 2021) and Thorakkattu, Khanashyam (Thorakkattu et al., 2022) have also shown that postbiotics contain essential vitamins, including vitamin B12, vitamin B2, vitamin B6, folic acid (vitamin B9), and vitamin K, which can be produced by some probiotic strains during fermentation of prebiotics.

#### 2.1.1 Bioactive peptides and their biotherapeutic potential for chronic disease management

BAPs are short chains of amino acids that are derived from proteins through enzymatic hydrolysis, fermentation, or digestion. BAPs typically consist of 2 to 20 amino acids and the specific sequence of amino acids in the peptide chain is crucial for determining its biological activity (Karami and Akbari-Adergani, 2019; Rizwan et al., 2023). Essential and non-essential amino acids mostly glycine, Isoleucine, leucine, proline, arginine, Valine, Tyrosine, and lysine are common in BAPs.

ACE inhibitors peptides work by preventing the conversion of angiotensin I to angiotensin II, a strong vasoconstrictor, which helps to lower blood pressure (BP) and offers vascular protection **Table 1**. Additionally, these inhibitors enhance the availability of bradykinin, a substance known for its vasodilatory effects **Table 1**. Bioactive peptides derived from *L. amylovorus* have shown promising effects in modulating lipid metabolism and preventing obesity-related disorders **Table 1**. These peptides can reduce lipogenesis by inhibiting lipogenic enzymes and gene expression in hepatocytes and adipocytes (Udenigwe and Rouvinen-Watt, 2015).

**Table 1:** Bioactive peptides and other postbiotic components biofunctional properties at *in vitro* and *in vivo* studies.

Type of probiotics	Postbiotics type	Type of Study	Biofunctional role	References
*L. brevis, L. helveticus, and L. paracasei*	Angiotensin-converting enzyme (ACE) inhibitory peptides	*In vitro*	Lowering blood pressure	(Ahn et al., 2009; Gonzalez-Gonzalez et al., 2013)
*L. amylovorus*	Lipid metabolism modulators peptides, and Antioxidant peptides	*In vivo*	Anti-obesity, prevent and treat dyslipidemia	(Bhat and Bajaj, 2019)
*Lactobacillus* spp	Butyric, Propionic, and Acetic acids	*In vitro*	Reduce obesity and diabetes	(Ibrahim et al., 2021)
*B.adolescentis, L.casei*,	Butyrate, Acetate	*In vitro*	Protect obesity and Type 2 diabetes	(Arora and Tremaroli, 2021) (Saravanakumar et al., 2021)
*B.adolescentis*	Butyrate	*In vitro*	Anti-inflammatory improve the integrity of the gut barrier	(Kim et al., 2022) (Singh et al., 2023)
*L.casei, L.fermentum, B.adolescentis*	Butyrate, Acetate, Propionate	*In vitro*	Inhibits cardiovascular disease and protect colorectal cancer, Maintain gut microbiota.	(Facchin et al., 2024)
*L. plantarum, B. longum*	Exopolysaccharides	*In vitro*	Antioxidant effect, anti-inflammatory and anti-type 2 diabetes, Anti-cancer	(Kwon et al., 2020) (Liang et al., 2024) (Inturri et al., 2017)
*L. plantarum*	Bacteriocins	*In vitro*	Anti-bacterial, Anti-inflammatory, Anti-obesity & anti-diabetes	(Dias, 2023)
*L. casei, L. fermentum* *B. adolescentis, B. Longum.*	Superoxide Dismutase, Catalase, Glutathione Peroxidase	*In vitro* and *In vivo*	Antioxidant, Reducing intestinal inflammatory	(Ahmad et al., 2022; Song et al., 2023)

The production of BAPs through fermentation is a complex process influenced by microbial strains, fermentation conditions, and type of protein substrate. Probiotic strains, particularly lactic acid bacteria (LAB), play a crucial role in this process. LAB possess proteolytic enzymes that break down food proteins into smaller peptides. During fermentation, LAB also metabolize carbohydrates, producing lactic acid, which lowers the environmental pH. This acidic condition enhances the activity of proteolytic enzymes, facilitating the hydrolysis of proteins into BAPs (Hati et al., 2014; Ter et al., 2024). The specific profiles of BAPs produced depend on the protein source and the LAB strains used, as the amino acids released during proteolysis vary across different substrates and microbes.

In addition to fermentation, enzymatic hydrolysis using specific proteases can be employed to produce BAPs. This technique can be used independently or in combination with fermentation to increase peptide yield and diversity (Wen et al., 2023; Ter et al., 2024).

Once produced, BAPs exhibit a wide range of health benefits. They include antimicrobial peptides such as nisin, pediocin, and plantaricin, which combat microbial infections (Wen et al., 2023; Setiarto and Anshory, 2024). Antioxidant peptides like Leucine-Leucine-Proline (LLP) and Valine-Tyrosine-Proline (VYP) scavenge free radicals, reducing oxidative stress (Abdulhussain Kareem and Razavi, 2020; Setiarto and Anshory, 2024). Anti-inflammatory peptides, such as Valyl-Prolyl-Proline (VPP) and Isoleucine-Proline-Proline (IPP), help reduce inflammation (Ahansaz et al., 2023; Setiarto and Anshory, 2024). Furthermore, anti-inflammatory peptides such as Valyl-Prolyl-Proline (VPP) and Isoleucine-Proline-Proline (IPP) contribute to inflammation reduction (Ahansaz et al., 2023; Setiarto and Anshory, 2024), Notably, VPP and IPP also act as antihypertensive agents by inhibiting angiotensin-converting enzyme (ACE), which prevents the conversion of angiotensin I to angiotensin II, a potent vasoconstrictor. This action lowers blood pressure and enhances vascular protection (Paramithiotis et al., 2022; Ter et al., 2024). Both VPP and IPP are significant for managing blood pressure and inflammation, making them valuable in dietary interventions for cardiovascular health.

Additionally, ACE inhibitory peptides improve the availability of bradykinin, a vasodilatory substance, further contributing to blood pressure regulation and cardiovascular health (**Table 1**). Bioactive peptides derived from *Lactobacillus amylovorus* have shown potential in modulating lipid metabolism and preventing obesity-related disorders. These peptides reduce lipogenesis by inhibiting lipogenic enzymes and suppressing the expression of lipogenic genes in hepatocytes and adipocytes (Udenigwe and Rouvinen-Watt, 2015).

#### 2.1.2 Shorts chain fatty acids and their biotherapeutic potential

**Figure 1:**
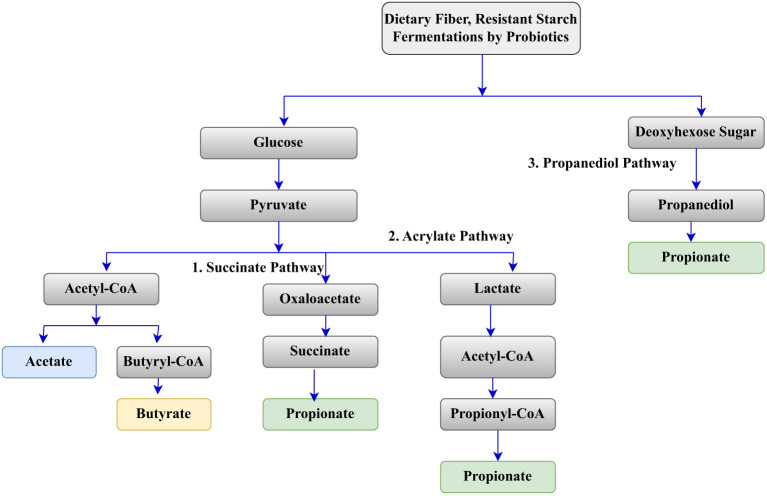
SCFAs production pathway.

SCFAs are fatty acids with fewer than six carbon atoms, primarily produced during the fermentation of dietary fibers by gut microbiota, particularly probiotics as shown in **Figure 1**. The most common SCFAs include acetate, propionate, and butyrate, which play significant roles in gut health and overall metabolic functions. Probiotics, ferment non-digestible carbohydrates (dietary fibers) into simpler sugars, which are then converted into SCFAs. Probiotic strains significantly impact the quantity and type of SCFAs produced during fermentation. The study of (Fernando et al., 2018) and (Farooq et al., 2013) showed that *Lactobacillus rhamnosus* and *Bifidobacterium bifidum* have been shown to effectively produce acetate and butyrate from fermentation of dietary fibers. The type of dietary fiber used as a substrate for fermentation has also affects SCFAs type and yields. The study of (Farooq et al., 2013) indicate that total dietary fiber (TDF) generally leads to higher SCFA production compared to soluble or insoluble fibers.

SCFAs, are crucial for gut health as they serve as a primary energy source for colonocytes, helping to maintain the integrity of the gut barrier and promoting the production of mucin, which protects the intestinal lining (LeBlanc et al., 2017; Chang et al., 2021). Additionally, SCFAs exhibit anti-inflammatory effects by modulating immune responses and reducing inflammation in the gut, with butyrate specifically down-regulating pro-inflammatory cytokines (Asarat et al., 2015). They also play a significant role in metabolic regulation, influencing energy metabolism, appetite, and fat storage, which can aid in weight management and overall metabolic health (LeBlanc et al., 2017). Furthermore, SCFAs contribute to microbiota modulation by inhibiting pathogenic bacteria and fostering the growth of beneficial species (Chang et al., 2021; Marnpae et al., 2024), thereby supporting a healthy gut microbiome. The type of probiotic, the substrate used, and fermentation conditions significantly influence SCFA production, which in turn offers various health benefits, particularly for gut health and metabolic regulation.

Bacterial lipids are integral to human health, significantly influencing gut microbiota and metabolic processes. Notably, sphingolipids and polyunsaturated fatty acids (PUFAs) serve essential physiological functions and offer various health benefits, underscoring their relevance in dietary and therapeutic settings (Sugawara, 2022; Wang X. et al., 2024). Bacterial sphingolipids, which are characterized by odd chain lengths, play a role in cell differentiation and immune responses, with the potential to migrate from the gut to other organs (Bai et al., 2023). Their interaction with dietary sphingolipids highlights a complex relationship that enhance host health. PUFAs, such as eicosatetraenoic acid (EPA) and docosahexaenoic acid (DHA), are crucial for their anti-inflammatory and anti-tumor properties (Yamashita et al., 2021), and microorganisms can produce these beneficial fatty acids, providing a sustainable source for dietary supplements (Béligon et al., 2016).

In addition to these lipid classes, the influence of bacterial outer-membrane vesicles (OMVs) and extracellular vesicles (EVs) on host health is an emerging area of research with implications for various diseases. Both are nanosized vesicles released by bacteria, containing bioactive molecules that can interact with host cells (Meng et al., 2024; Razim et al., 2024). OMVs is a nanoscale phospholipid bilayer particles released by bacteria, encapsulate a variety of biomolecules, including lipids, proteins, and nucleic acids (Zwarycz et al., 2020; Ruiz-Moreno et al., 2024). These vesicles play a pivotal role in intercellular communication, immune modulation, and the delivery of bioactive lipids to host cells (Ghadami and Dellinger, 2023). For instance, OMVs can transport bacterial sphingolipids and other lipid mediators, potentially influencing host immune responses and gut homeostasis. They have also shown potential in cancer treatment by delivering therapeutic agents directly to tumor sites, minimizing systemic toxicity, carry specific antigens and immunomodulatory compounds, and enhancing immune responses against cancer cells (Meng et al., 2024).

Similarly, EVs, which are secreted by both Gram-positive and Gram-negative bacteria, are enriched in lipids that contribute to their structural integrity and functional roles in signaling (Melo-Marques et al., 2024; Razim et al., 2024) and nutrient exchange (Leiva-Sabadini et al., 2024; Melo-Marques et al., 2024). The lipid composition of these vesicles, including their unique lipid bilayer organization, is essential for their stability and ability to interact with host cells (Xavier et al., 2020).

The interplay between bacterial lipids and vesicles underscores their multifaceted role in human health. By facilitating the transport of bioactive lipids and mediating host-microbe interactions. OMVs and EVs expand the scope of bacterial lipid functions beyond their structural and metabolic roles. This emerging area of research highlights the potential of bacterial vesicles as therapeutic tools and diagnostic biomarkers in the context of gut health and metabolic diseases (Jalalifar et al., 2023).

#### 2.1.3 Exopolysaccharides and their biotherapeutic potential

EPS are complex, high-molecular-weight carbohydrates produced by various microorganisms. They can be produced through the fermentation of dietary fibers by probiotic bacteria, particularly LAB species. During this process, probiotics break down complex carbohydrates into simpler sugars, which are then converted into EPS. The type of probiotic strain, substrate composition, and fermentation conditions can influence the quantity and composition of the produced EPSs (Maftei et al., 2024; Manoharan et al., 2024).

EPS have promising results in modulating the immune-inflammatory response in IBD patients. Studies have shown that EPSs produced by Streptococcus mutans and *Lactobacillus acidophilus* can affect the metabolic activity and viability of human gingival fibroblasts, which are crucial for the progression of chronic periodontitis (Szkaradkiewicz-Karpińska and Szkaradkiewicz, 2021). The research conducted by Kwon et al. (2020) demonstrated that EPS derived from *Lactobacillus plantarum* may serve as a natural therapeutic agent for inflammatory diseases. This is achieved by inhibiting pro-inflammatory mediators such as IL-6, TNF-α, and COX-2, suppressing TLR4 expression and its activation by LPS, and regulating the MAPK and NRF2/HO-1 pathways, which ultimately reduces oxidative stress.

EPS regulate pro-inflammatory cytokines while promoting the production of anti-inflammatory mediators (Zampieri et al., 2020; Manoharan et al., 2024), thereby influencing the activity and differentiation of immune cells, including T cells and regulatory T cells, to maintain immune homeostasis (Manoharan et al., 2024).

Additionally, EPS inhibit the growth of pathogenic microorganisms, which helps prevent infections and supports a healthy gut microbiome (Maftei et al., 2024; Manoharan et al., 2024). Furthermore, they serve as a vital food source for beneficial gut bacteria, promoting their growth and proliferation (Maftei et al., 2024). Their anti-inflammatory, immunomodulatory, and antimicrobial properties make them attractive alternative for biotherapeutic of chronic disease.

EPSs are produced by microorganisms as a protective layer, aiding in biofilm formation and providing resistance to environmental stresses. They are composed of various monosaccharides and can exhibit diverse structures and properties depending on the sugar unit and the producing microorganisms’ strain as well as environmental conditions (Nemati and Mozafarpour, 2024). EPS) are classified into homo-EPS, which are large (greater than 1000 kDa) and composed of a single type of sugar residue, and hetero-EPS, which are smaller (ranging from 100 to 1000 kDa) and consist of various types of sugar residues (Lu et al., 2023). Probiotics can utilize various substrates, including lactose, sucrose, and inulin, to synthesize EPS and addition of some substrate like inulin as fermentation substrate has been shown to enhance EPS biosynthesis (Guan et al., 2023).

## 3 Mechanisms of action of postbiotics

One of the key mechanisms by which postbiotics exert their effects is through the enhancement of gut barrier function. They achieve this by modulating tight junctions and promoting mucin production. Postbiotics regulate the expression of critical tight junction proteins, such as occludin and claudin, via the activation of signaling pathways like the PI3K/Akt pathway (**Table 2**). This process strengthens the intercellular connections within intestinal epithelial cells, thereby fortifying the integrity of the gut barrier (Tavalali et al., 2001). Additionally, they stimulate the production of mucins, protective glycoproteins secreted by goblet cells, by enhancing the expression of the MUC2 gene via the NF-κB signaling pathway. This increased mucin production contributes to a robust gut barrier, offering protection against pathogens and inflammation (Om et al., 2024).

**Table 2:** Common postbiotics biotherapeutic mechanisms of actions.

Mechanism of Action	Effects	References
Activation of PI3K/Akt pathway	Increased expression of tight junction proteins (occludin, claudin)	(Tavalali et al., 2001)
Activation of NF-κB pathway	Increased mucin production (MUC2 gene expression)	(Om et al., 2024)
Activation of Toll-like receptors (TLRs)	Balanced production of pro- and anti-inflammatory cytokines	(Mosca et al., 2019; Chen et al., 2024)
Interaction with G-protein-coupled receptors (GPCRs) (FFA2, FFA3)	Anti-inflammatory effects	(Liu et al., 2024; Wang X. et al., 2024)
Inhibition of NF-κB pathway	Reduced inflammation, particularly relevant in IBD and metabolic disorders	(Liang and Xing, 2023)
Modulation of gut microbiome	Increased SCFA production, restored and enhance microbial diversity, promoted beneficial bacteria	(Zhong et al., 2022; Liu et al., 2024)
Support of *Akkermansia muciniphila*	Enhanced gut barrier function, reduced inflammation, improved metabolic health	(Wang et al., 2021; Cheng et al., 2022; Wade et al., 2023; Zheng et al., 2023)

They modulate the immune system, which is closely linked to gut barrier function. They activate Toll-like receptors (TLRs), their activation leads to the production of both pro-inflammatory and anti-inflammatory cytokines, thereby maintaining immune balance and gut homeostasis (Mosca et al., 2019; Chen et al., 2024). Furthermore, postbiotics interact with G-protein-coupled receptors (GPCRs), such as FFA2 and FFA3, which mediate anti-inflammatory effects, contributing to a healthier gut environment (Liu et al., 2024; Wang X. et al., 2024).

Postbiotics demonstrate considerable biotherapeutic potential, particularly in inhibiting inflammatory pathways. A key mechanism involves the modulation of the nuclear factor kappa-light-chain-enhancer of activated B cells (NF-κB) pathway, a critical regulator of inflammation (**Table 2**). Postbiotics can suppress this pathway by preventing NF-κB translocation to the nucleus, thereby reducing the expression of pro-inflammatory cytokines. This anti-inflammatory action is especially relevant in managing chronic inflammatory conditions such as inflammatory bowel disease (IBD) and metabolic disorders (Liang and Xing, 2023).

As shown in **Table 2**, postbiotics play a significant role in modulating the gut microbiome, restoring and enhancing the diversity of gut microbes within the gastrointestinal tract (Nagpal et al., 2018; Bianchi et al., 2019). Postbiotic bioactive compounds such as SCFAs, vitamins, BAPs, and other bioactive compounds, serve as substrates or signaling molecules that selectively support the growth of beneficial microbial species. This microbial community can be disrupted by various factors, including poor dietary habits, antibiotic use, and certain diseases (Huang et al., 2021; Popov et al., 2024). Furthermore, postbiotics promote the growth of beneficial bacteria, such as *Bifidobacterium* spp. and *Lactobacillus* spp., which are recognized for their positive impact on gut health (Nagpal et al., 2018; Bianchi et al., 2019; Mao et al., 2019). These bacteria contribute to the enhancement of the gut barrier and the production of short-chain fatty acids (SCFAs) such as acetate, propionate, and butyrate (Nagpal et al., 2018; Bianchi et al., 2019; Mao et al., 2019).They can also modulate immune responses by promoting the differentiation of regulatory T cells (Tregs) and influencing cytokine production. A study by Xu, Wu (Xu et al., 2023) demonstrated that postbiotics derived from *Saccharomyces boulardii* significantly modulated inflammatory responses in a mouse model of ulcerative colitis. The administration of these postbiotics resulted in increased levels of anti-inflammatory cytokines (such as IL-10) and decreased levels of pro-inflammatory cytokines (including IL-1β, IL-6, and TNF-α), highlighting their role in restoring immune balance and reducing inflammation.

**Figure 2:**
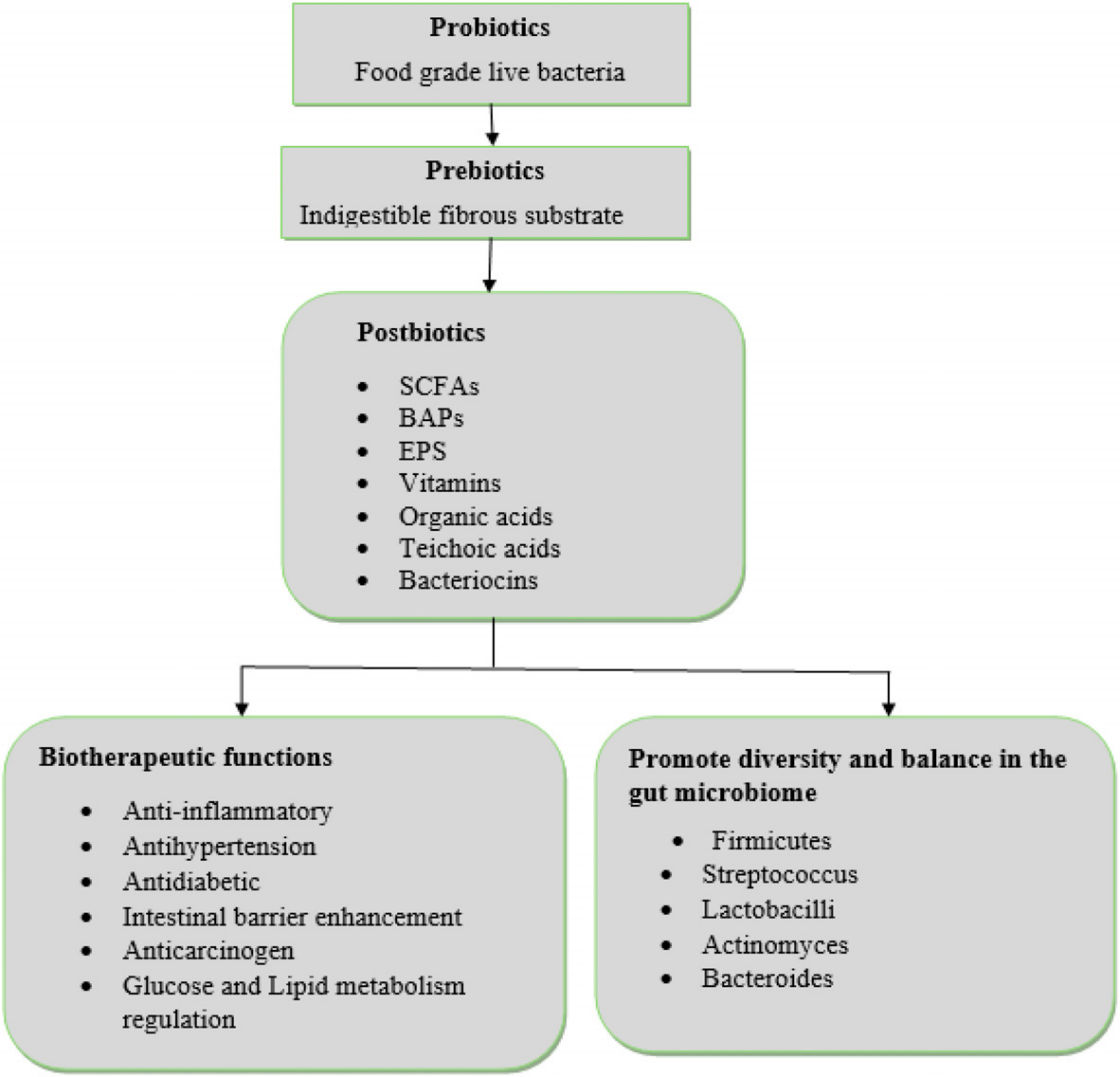
Postbiotics mechanisms of actions. SCFAs, Short-Chain Fatty Acids; BAPs, Bioactive Peptides; EPS, Exopolysaccharides.

As shown in **Figure 2**, postbiotic exert their influence on chronic diseases through two primary mechanisms. Firstly, they directly contribute to biotherapeutic functions, such as anti-inflammatory and antioxidant effects. Secondly, they indirectly modulate the gut microbiota, promoting a favorable composition and diversity, which in turn, ameliorates chronic disease management. Additionally, postbiotics strengthen the integrity of the intestinal epithelial barrier, a critical factor in preventing pathogen translocation and maintaining gut health. An *in vitro* study by Liu, Jiang (Liu et al., 2024) demonstrated that postbiotic administration in alcohol-induced chronic liver disease significantly increased the expression of tight junction proteins, reducing intestinal permeability and preventing the systemic circulation of harmful substances. Additionally, research by Hijová (2024) indicated that postbiotics derived from *Lactobacillus plantarum* exhibit significant antioxidant activity, which is particularly beneficial in chronic diseases characterized by oxidative damage, such as cardiovascular diseases and neurodegenerative disorders.

Postbiotics can provide SCFAs and other metabolites that serve as energy sources for intestinal epithelial cells and gut microbiota. This can support intestinal mucosal healing and overall gut health. The study by Hosseini, Abbasi (Hosseini et al., 2023) demonstrated that *in vitro* produced SCFAs, such as butyrate, can reduce inflammation, improve gut health, and inhibit the activation of inflammatory pathways, thereby contributing to their protective effects in chronic diseases. Furthermore, research by Ying, Mao (Ying et al., 2023) on postbiotics in rheumatoid arthritis indicated that they could modulate inflammatory pathways and reduce the expression of inflammatory mediators. This suggests that postbiotics may serve as a viable adjunctive therapy for rheumatoid arthritis by influencing immune processes and bone metabolism.

### 3.1 Major challenges to utilize postbiotics to improve human health

Utilizing postbiotics for the enhancement of human health, particularly in the context of chronic diseases, presents some critical challenges. Despite growing interest, the body of research on postbiotics is still relatively small compared to probiotics and prebiotics. More extensive clinical trials are needed to establish the health benefits, human clinical trials, and optimal dosages of postbiotics. This scarcity of human-focused research restricts the ability to generalize findings and comprehensively assess the full potential and safety of postbiotics applications in human populations (Vallianou et al., 2020; Eslami et al., 2024). Without extensive clinical trials, it is difficult to establish effective treatment protocols or understand the long-term impacts of postbiotic consumption.

The variability in study designs is another major hurdle. There exists significant inconsistency in the formulations, doses, and types of postbiotics utilized across different studies. This heterogeneity complicates the comparison of results and limits the ability to draw definitive conclusions regarding the efficacy and safety of postbiotics (Vallianou et al., 2020; Mehta et al., 2023). As a result, creating standardized protocol for its utilization face challenges.

While postbiotics are generally regarded as safe, there is a pressing need for more comprehensive safety evaluations, particularly concerning their long-term use and effects in specific population groups, such as allergic, immunocompromised individuals (Vallianou et al., 2020; Mehta et al., 2023). Ensuring that postbiotics do not pose any adverse effects in vulnerable populations is crucial for their broader acceptance and use in clinical settings.

A significant challenge lies in the limited information and understanding on the mechanisms by which postbiotics exert their beneficial effects. More research is needed to elucidate how various components of postbiotics interact with human physiology and contribute to health improvements (Li et al., 2021; Wu et al., 2023). A deeper understanding of these mechanisms is essential for optimizing postbiotic formulations and enhancing their therapeutic applications.

The lack of standardized guidelines for the production and quality control of postbiotics poses a barrier to their widespread adoption. Inconsistent product quality and efficacy can undermine trust among healthcare providers and patients (Scarpellini et al., 2021; Mehta et al., 2023). Establishing clear regulatory frameworks and quality assurance protocols is necessary to ensure that postbiotic products meet safety and efficacy standards.

Translating findings from preclinical studies into clinical practice presents a substantial challenge. There is a pressing need for high-quality, large-scale randomized controlled trials to validate the efficacy of postbiotics in the treatment of chronic diseases and to determine optimal dosing regimens (Wu et al., 2023; Eslami et al., 2024). Such rigorous investigations are crucial for establishing the clinical relevance of postbiotics and for enabling their incorporation into standard treatment protocols.

## 4 Future perspective

The investigation into the therapeutic potential of postbiotics is currently in its nascent phase, highlighting the need for extensive research to determine their efficacy, optimal dosages, bioavailability, storage stability, and long-term health effects. Future studies should prioritize elucidating the specific mechanisms through which different postbiotic compounds exert their beneficial effects, as well as exploring their potential applications in a variety of chronic conditions, such as obesity, diabetes, cancer and cardiovascular diseases. This deeper understanding could pave the way for more targeted and effective therapeutic strategies. Furthermore, incorporating postbiotics into functional foods and dietary guidelines represents a promising avenue for enhancing public health initiatives. By promoting the consumption of postbiotic-rich foods, health authorities could encourage preventive care and ultimately improve health outcomes, particularly among populations at heightened risk for noncommunicable diseases. Such integration could foster a proactive approach to health management, emphasizing the importance of diet in disease prevention and overall wellness.

## 5 Conclusions

Postbiotics are bioactive compounds generated from the metabolic processes of probiotics, offering health benefits without the need for live microorganisms. This characteristic makes them particularly appealing, as they retain the advantages of probiotics while alleviating concerns about the stability of live bacteria. The rise of chronic diseases, such as cardiovascular diseases, diabetes, and obesity, poses significant health challenges globally, especially in low- and middle-income countries, where they contribute to high morbidity and economic burdens. In this context, postbiotics present a promising biotherapeutic option for managing chronic diseases through mechanisms like immune modulation, gut barrier enhancement, and antioxidant activity. Key components of postbiotics include SCFAs, BAPs, and EPS, which play essential roles in metabolic health, inflammation regulation, and gut health. Collectively, these attributes highlight the potential of postbiotics in disease prevention and promoting overall metabolic wellness.
